# Observing the World Through Your Own Lenses – The Role of Perceived Adaptability for Epistemological Beliefs About the Development of Scientific Knowledge

**DOI:** 10.3389/fpsyg.2018.01006

**Published:** 2018-06-20

**Authors:** Ronny Scherer, Øystein Guttersrud

**Affiliations:** ^1^Centre for Educational Measurement, Faculty of Educational Sciences, University of Oslo, Oslo, Norway; ^2^The Norwegian Centre for Science Education, University of Oslo, Oslo, Norway

**Keywords:** adaptability, epistemological beliefs, knowledge development, measurement invariance, moderated non-linear factor analysis

## Abstract

Students are exposed to vast amounts of information and knowledge that is rapidly changing. This exposure requires them to be adaptive, that is, to constantly adjust their thinking, behavior, and even their affect to successfully solve information-rich and knowledge-lean problems. Considering these developments, the purpose of the present study is twofold: First, it is aimed at exploring the link between students’ beliefs about their adaptability in an ever-changing world and their beliefs about the changing nature of scientific knowledge, thus linking two educationally relevant belief systems. Second, this study further explores validity issues related to the well-established and commonly used “Epistemological Beliefs about the Development of Scientific Knowledge (EBDE)” scale. Performing structural equation modeling on a large-scale data set of 1,662 Norwegian tenth-grade students, we estimated the correlations among different aspects of adaptability (i.e., cognitive-behavioral and affective-emotional adaptability) and EBDE. Moving beyond these correlations, we tested whether students’ perceived adaptability had an impact on the functioning of EBDE items by means of moderated factor analysis. Our analyses revealed that adaptability was associated with sophisticated EBDE in science, and the EB scale functioned differently with respect to different adaptability scores. The results of this study indicate that students perceive the development of scientific knowledge through the lenses of their own adaptability. Furthermore, the differential functioning of the EBDE scale challenges its validity.

## Introduction

There is no doubt, the world is developing. In fact, knowledge, information, and technology are rapidly advancing, making it harder for us to keep up with the latest developments and insights. This development constantly exposes us to situations, in which we have to adjust our thinking and behavior to novel, uncertain, and changing situations across almost all areas of our lives (Winthrop and McGivney, 2016)—for instance, when dealing with disruptions in our daily commuter routes (Harford, 2012) or when making up our minds about climate change (McClanahan and Cinner, 2011). But how prepared do we feel to cope with such change? Indeed, our beliefs about how adaptable we are play a major role for performance and learning in complex problem situations (Barak and Levenberg, 2016).

Our information society demands to constantly adjust one’s thinking, behavior, affect, and emotions to novel and changing situations—hence, this capacity, which is often called “adaptability,” has gained interest in educational and psychological research (Pellegrino and Hilton, 2012; Martin et al., 2013). More precisely, an increasing body of literature reports on the relevance of students’ beliefs about how adaptable they are for academic success, well-being, buoyancy, self-control – the list of important outcome variables is growing (e.g., Pulakos et al., 2002; Gloster et al., 2011; Martin et al., 2015; Collie et al., 2016). At the same time, the demands to adapt to novelty, uncertainty, or changes interfere with the way we perceive these demands. Put differently, our beliefs about the changing and developing nature of knowledge, information, or technology – so-called “epistemological beliefs” – play another critical role for success in many areas (Trautwein and Lüdtke, 2007; Green and Hood, 2013). In the pursuit of disentangling what might determine adaptive expertise, two questions remain largely unanswered: To what extent does adaptability correspond to the way we view this rapidly changing world? and to what extent are perceived adaptability and epistemological beliefs about the developing and changing nature of knowledge related? Knowledge about this relation clarifies whether two seemingly distinct belief systems—self-beliefs and epistemological beliefs—are linked. Moreover, it provides educators with possibilities to influence the one by fostering the other. We notice that adaptability surfaces in several life situations; hence, the questions we are posing here are not restricted to certain domains or contexts.

Using assessments of cognitive flexibility—a concept that taps the cognitive aspects of adaptability, yet does not include affective-emotional aspects—some researchers uncovered a positive relation between flexibility and epistemological beliefs (Kienhues and Bromme, 2011; Roex et al., 2011). Elen et al. (2011) argued that the two concepts are closely connected and can even considered to be indicators of each other. Moreover, it has not yet become clear to what extent the measurement of epistemological beliefs is sufficiently invariant across different levels of flexibility. This question concerns the validity of the measure (Pellegrino et al., 2016). Knowledge about the invariance of epistemological beliefs measures along the continuum of adaptability provides test developers and assessment specialist with information about the functioning of the measures and the cautions associated with the interpretation of the resultant test scores (Scherer, 2017).

This paper seeks to clarify the link between adaptability and epistemological beliefs about the development of scientific knowledge (EBDE) by examining (a) the correlations between perceived adaptability – including its cognitive-behavioral and affective-emotional aspects – and EBDE; (b) the extent to which the most commonly used measurement of EBDE—Conley et al.’s (2004) Science Epistemological Beliefs Scale (see also Liu, 2010; Tsai et al., 2011; Kampa et al., 2016)—is affected by individual differences in adaptability. We adopt a moderated factor analysis approach and include further variables representing students’ background.

## Theoretical Framework

This section is organized as follows: First, we provide a brief review of the existing bodies of literature on students’ perceived adaptability and next EBDE. This brief review includes the conceptualization and aspects of the two constructs and describes the theoretical basis for the relation between perceived adaptability and EBDE.

### Perceived Adaptability

#### Construct Definition

Adaptability has many facets: First and foremost, it refers to the capacity to adjust one’s thinking, behavior, emotions, and affect to changing, novel, and uncertain^1^ situations (VandenBos, 2007). In light of this conceptualization, adaptability comprises cognitive, metacognitive, volitional, motivational, and even emotional elements. Mayer (2014), for instance, emphasized the cognitive and metacognitive processes of adaptability and summarized them under the umbrella of what he called ‘adaptive problem solving,’ that is, “a form of problem solving that requires a series of problem reformulations or continual reevaluation of problem formulations in light of changing conditions. In short, adaptive problem solving occurs when a problem solver continually revises his or her problem representations (and the corresponding solution plan) in light of the changes in the problem situation” (p. 153). Several problem situations in our daily lives require us to adapt, perhaps because our strategies to solve them did not work out or because the problem itself or the information attached to it changed. In fact, we are constantly required to perform adaptive problem solving and therefore engage in the cognitive and metacognitive processes Mayer (2014) mentioned. Acknowledging the importance of these processes and their relevance not only in everyday-life situations but also in work settings has led to the inclusion of adaptability in existing twenty-first century skills frameworks (Chan, 2014; Soulé and Warrick, 2015). In these frameworks, however, the status of adaptability may differ substantially: On the one hand, it might be considered a form of problem solving or critical thinking and therefore a construct that taps cognitive and metacognitive thinking processes (Mayer, 2014; Scherer, 2015; Barak and Levenberg, 2016). On the other hand, adaptability might be considered a personality trait that taps the willingness and openness to adjust our thinking and behavior to changing, novel, and uncertain situations (Martin et al., 2012b; Kaufman et al., 2016). Even further, in their almost exhaustive taxonomy describing the constituents of the composite construct adaptability, Pulakos et al. (2000) identified its core elements: the abilities to solve complex problems, to deal with uncertain problem situations, and to adapt to emotionally challenging situations or to cultural experiences. These different but related aspects capture the complexity of the construct, indicate the presence of possible sub-factors, and express the diversity of demands that come along with novel, uncertain, and changing situations.

#### Dimensions of Adaptability

The current perspective on adaptability suggests that cognitive-behavioral and affective-emotional aspects can be differentiated (Martin and Rubin, 1995; Pulakos et al., 2000). Putting this perspective to test, Martin et al. (2012b, 2013) conducted multiple studies in which they specifically searched for evidence on the distinction between cognitive-behavioral and affective-emotional aspects of adaptability. Martin and colleagues developed a self-report scale measuring students’ perceived adaptability. The results were clear-cut across several student samples: Empirical evidence strengthened the hypothesized factor structure that distinguished between a cognitive-behavioral and affective-emotional adaptability; yet the correlation between the two aspects was high, *r* = 0.88. This structure was also invariant across educationally relevant groups, such as gender, age, and linguistic backgrounds (Martin et al., 2012b). These findings provide empirical evidence for the hypothesis that the two aspects of adaptability are distinct.

From a conceptual perspective, the delineation of cognitive-behavioral and affective-emotional dimensions of adaptability relates to different research traditions: The *cognitive* aspect of adaptation or adaptive behavior is closely linked to basic executive functions, such as cognitive flexibility, working memory updating, and inhibitory control (Diamond, 2013). In fact, these functions form the basis for processing new information (updating), shifting between tasks and problem situations (flexibility), and focusing on relevant challenges and information (inhibition). Even further, students’ skills to adapt their thinking about scientific problems or concepts manifests in what is often referred to as “conceptual change,” that is, the adaptation of initial, everyday life conceptualizations of scientific phenomena to more scientific information and evidence that challenges them. Vosniadou and Kampylis (2013) argue that cognitive flexibility and conceptual change go hand in hand. Besides the linkage between well-established, cognitive concepts (i.e., conceptual change and executive functions) and cognitive-behavioral adaptability, there is also a remarkable overlap between the *affective-emotional* dimensions of adaptability and cognate concepts. Facing new situation and problems can become rather stressful for some people—Pulakos et al. (2000) thus considered emotional adaptation critical for problem-solving success. For instance, the skills to adjust one’s emotions in sudden, perhaps stressful situations are closely related to coping flexibility and psychological adjustment (Cheng et al., 2014). Strengthening the claim that these skills interfere with the cognitive-behavioral dimension of adaptability, Lopes et al. (2012) emphasize their relevance in social context, such as classrooms. Overall, both aspects of adaptability are critical to successful learning, problem solving, and mental health (Collie and Martin, 2017; Martin et al., 2017).

#### Individual Differences

Adaptability correlates significantly with relevant personality traits, academic and non-academic well-being including self-esteem, life satisfaction, sense of meaning and purpose, and emotional stability, implicit theories, and academic outcomes (e.g., school engagement), supplementing the creation of a validity argument of the existing Martin et al. Adaptability Scale (Martin et al., 2012b, 2013, 2015). Martin et al. (2012b) further observed variation in the correlations between cognitive-behavioral and affective-emotional adaptability across different samples. Among the findings presented above, one merits evaluation: The invariance of the perceived adaptability measure across students with different language backgrounds (Martin et al., 2012b). More precisely, students whose mother tongue was not English^2^ perceived themselves as more adaptable than native students. A number of later studies that applied performance-based rather than self-report measures of adaptability in problem solving situations have found similar effects (Martin et al., 2012a; Sonnleitner et al., 2014). In their meta-analysis, Adesope et al. (2010) identified a significant covariation between bilingualism and cognitive flexibility, thus strengthening the hypothesis that students with immigration status might be more adaptable than native students. Attempting to explain this observation, Martin et al. (2012a) argued that, immigrant students are exposed more frequently to situations triggering the need for adaptation (see also Martin et al., 2012a).

To this end, the present study will draw from the insights gained from existing empirical studies on adaptability by building on the distinction between cognitive-behavioral and affective-emotional adaptability and by examining potential differences between native and immigrant students in both the overall level of adaptability and in the measurement of the construct.

### Epistemological Beliefs About the Development of Scientific Knowledge

#### Construct Definition

Epistemology concerns the nature of human knowledge and its justification (Hofer and Pintrich, 1997). Along these lines, students’ epistemological beliefs refer to the views of their representations of scientific knowledge and what it means to know (Mason and Bromme, 2010). Epistemological beliefs are considered to be vital for learning and conceptual understanding within several domains and contexts (e.g., Buehl et al., 2002; Bråten and Strømsø, 2005; Trautwein and Lüdtke, 2007; Bromme et al., 2009; Mason and Bromme, 2010; Liu and Liu, 2011; Green and Hood, 2013).

#### Dimensions of Epistemological Beliefs in Science

The existing body of research concludes that these beliefs comprise a number of core aspects that tap different aspects of scientific knowledge (e.g., Conley et al., 2004; Trautwein and Lüdtke, 2007; Kampa et al., 2016). For instance, Conley et al. (2004) proposed four core aspects: Beliefs about how scientific knowledge develops and notions of its uncertainty, sources, and justifications. A number of studies across countries and age groups later empirically identified these aspects as sub-dimensions bringing multidimensionality into the data (see Tsai et al., 2011; Chen, 2012). More specifically, these dimensions describe beliefs in: (a) scientific knowledge as either being right or wrong, or the need to view scientific knowledge from multiple perspectives (Kampa et al., 2016); (b) the tentative nature of scientific knowledge owing to new evidence, changes in existing evidence, or new interpretations of existing evidence; (c) the subjective nature of scientific knowledge as residing in external, authoritarian sources, such as scientists or teachers (Conley et al., 2004); (d) the empirical nature of science adhering to the role of scientific investigations as means to justify claims, models, and hypotheses.

#### Individual Differences

The existing body of research suggests that epistemological beliefs are positively related to achievement in science (e.g., Conley et al., 2004; Chen, 2012; Kampa et al., 2016). Indeed, in the early publication of Conley et al.’s (2004) works, between-student variation could be explained by variation in achievement scores. The list of further constructs that are significantly related to these beliefs included implicit theories, self-beliefs, and the understanding of scientific concepts (Tsai et al., 2011; Chen, 2012; Kampa et al., 2016). Besides these, students’ background, for instance, indicated by their socioeconomic status, represents another source of individual differences (Conley et al., 2004). It is therefore important to consider background variables when examining the adaptability-epistemological beliefs link and the invariance of the epistemological beliefs measure (Scherer, 2017).

In the current study, we focus on students’ *epistemological beliefs about the development of scientific knowledge*; these beliefs reflect best the tentativeness and changing nature of scientific knowledge – aspects that play a significant role in adaptation and flexible thinking (Barak and Levenberg, 2016). Besides, the concepts of perceived adaptability and EBDE are well-aligned as their conceptualizations resonate with the notions of change and development.

### The Relation Between Perceived Adaptability and Epistemological Beliefs

**FIGURE 1:**
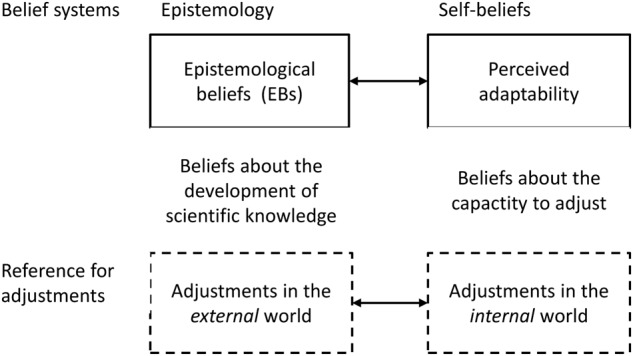
Conceptual model underlying the relation between perceived adaptability and EBDE.

While perceived adaptability is part of a belief system directed toward the self – the expected level of flexibility (self-beliefs)—EBDE reflect a belief system directed toward the external world—the individual’s ideas about the nature of scientific knowledge (epistemological beliefs). **Figure 1** illustrates this distinction between these two belief systems. While the first is based on the individual’s aggregated prior experiences, the latter is concerned with ideas founded on tentative consensus outside the self (Tsai et al., 2011; Kapucu and Bahçivan, 2015). At the same time, the two constructs are closely aligned – the notion of changes and tentativeness forms the unifying element (Elen et al., 2011). In the following, we briefly review the existing knowledge about the relation between these two belief systems.

A number of studies have examined the link between epistemological beliefs and self-beliefs, most of which conceptualized self-beliefs as self-efficacy or self-concept in learning and anchored them in theories of social cognition (Pajares and Schunk, 2001; Jansen et al., 2015). For instance, Mason et al. (2013) studied the relations among EBDE, achievement goal orientations, knowledge, and self-beliefs, and they provided evidence that high self-concept in science is linked to sophisticated EBDE across grade levels. Tsai et al. (2011) explored the link between EBDE and self-beliefs in science learning and found direct effects of epistemological beliefs on self-efficacy beliefs and indirect effects via students’ conceptions of science learning. These effects varied across the aspects of epistemological beliefs; only EBDE were positively related to self-efficacy beliefs via learning conceptions. Kapucu and Bahçivan (2015) further strengthened the idea that the relations among self-efficacy and epistemological beliefs differ with respect to the aspects of epistemological beliefs. They could not identify significant relations for the EBDE. In contrast, Chen (2012) showed that EBDE and self-efficacy in science correlated significantly and positively. Chen extended the measures of self-beliefs by measures of students’ mindsets and found that a flexible, growth-oriented mindset was associated with sophisticated EBDE. This finding further strengthens the idea that perceived adaptability and EBDE are related.

Roex et al. (2011) uncovered that medical students with sophisticated beliefs about the tentative nature of scientific knowledge were cognitively more flexible than those holding naïve beliefs. Kienhues and Bromme (2011) accentuated this finding by arguing that EBDE and self-beliefs about abilities are crucial for the processing of information in our digitalized world; as a matter of fact, the two concepts are considered indicators of cognitive flexibility. Elen et al. (2011) summarized that epistemological beliefs and cognitive flexibility are not only intertwined but indicators of each other. In light of these considerations, a positive relation between perceived adaptability and EBDE is, to some extent, expected.

Nonetheless, the relation between perceived adaptability and EBDE may be affected by the ways in which the constructs are measured. For instance, reports of the relation between the constructs may not only exist at the construct level but also at the item level (Millsap, 2011). Such item-level relations, however, conflate researchers’ ability to make valid inferences as they indicate that items function differently for students with different adaptability scores. This measurement perspective questions the validity of the EBDE measures. It brings forth the question as to whether the relation might also exist between specific parameters in the measurement model of EBDE and students’ perceived adaptability. In this sense, perceived adaptability is considered a potential covariate that might affect the functioning of EBDE items. If indeed differential item functioning (DIF) across ‘levels’ of the continuous variable perceived adaptability occurs, the validity of EBDE scores might be compromised (Millsap, 2011; Bauer, 2016). At the same time, the identification of such effects suggests that students view the development of scientific knowledge through the lenses of their own adaptability. In conclusion, the Adaptability—EBDE relation might exist at the construct *and* at the measurement level. Furthermore, in order to appropriately interpret correlations at the construct level, EBDE items displaying DIF with respect to perceived adaptability need to be identified (e.g., Curran et al., 2014). To study the link between adaptability and EBDE, we will focus on the well-established Adaptability Scale developed by Martin et al. (2012b) and the most commonly used Science Epistemological Beliefs Scale developed by Conley et al. (2004)—for these scales, sufficient evidence for reliability and selected aspects of validity (e.g., dimensionality, cross-cultural applicability) exists.

### The Present Study

As noted earlier, Stahl (2011) established a conceptual link between EBDE and cognitive flexibility, and further argued that both the stability and variability of epistemological beliefs may depend on a person’s perceived or enacted cognitive flexibility. The empirical evidence surrounding the assumed link between the two concepts – EBDE and cognitive flexibility – is, however, only limited, as Stahl (2011) pointed out. The present study consequently puts to test this assumption by shedding light on the relation between perceived adaptability – a construct that comprises perceived cognitive-behavioral and affective-emotional flexibility (as part of the self-beliefs system) – and beliefs about the changing nature of scientific knowledge (as part of the epistemological beliefs system). Besides examining the proposed relation between these constructs empirically, we extend Stahl’s argumentation to the measurement level. More precisely, we do not only examine the extent to which EBDE and adaptability are correlated at the *construct level*, but also study whether different adaptability scores affect the functioning of the measurement of EBDE (*measurement level*). This perspective provides further insights into the potential mechanisms that may underlie the relation between perceived adaptability and EBDE, and considers the potential differential functioning of the EBDE measure as a function of adaptability. The latter is of relevance when creating a validity argument of the proposed relation between the two constructs. These two perspectives –the construct-level and the measurement-level perspective – will also be explored with respect to students’ immigration status. Given that previous research uncovered effects of immigration background on adaptability and students’ proficiency in problem solving (Martin et al., 2012a; Sonnleitner et al., 2014), and researchers encouraged investigating these effects for EBDE (Conley et al., 2004; Kampa et al., 2016), we include this covariate in our modeling approach. In sum, the present study focuses on the following research questions (RQs):

(1) ***Construct level:***
  (a) To what extent are students’ epistemological beliefs about the development of scientific knowledge and their adaptability related?
  (b) To what extent are these relations subject to differences across students’ immigration background?
(2) ***Measurement level:***
  (a) To what extent does the measurement of students’ epistemological beliefs about the development of scientific knowledge show invariance along the continuum of the latent variable perceived adaptability?
  (b) Can measurement invariance be established across the interactions between perceived adaptability and immigration background?

## Materials and Methods

### Sample and Procedure

The present study was based on a sample of *N* = 1,662 Norwegian tenth-grade students (50.9% girls) in 90 lower secondary schools. Students’ average age was 15.4 years (*SD* = 0.5 years) and ranged between 15 and 16 years. In total, 11.2% of the entire sample reported that they were first- or second-generation immigrants, 84.2% indicated a native Norwegian background, and 4.5% did not respond to the questions about their status. In the current study, students worked on a questionnaire which comprised self-report scales of epistemological beliefs in science, perceived adaptability, and background information such as their immigration status. The questionnaire was computer-based, and we used the schools’ computer facilities to administer the assessments. In total, the test session took 90 min, and the resulting data were transferred online to a secure server. The entire dataset was anonymized such that students could not be identified in person.

### Measures

#### Epistemological Beliefs

Students’ epistemological beliefs about the changing nature of scientific knowledge were assessed by a Norwegian version of the commonly used ‘*Development of Scientific Knowledge*’ subscale of the Conley et al. (2004) EBDE measure. Liu (2010) argues that this Conley et al.’s (2004) measure has become the most prominent for the assessment of epistemological beliefs in science. As for other EBDE subscales, evidence supporting both the reliability and validity of this scale has been provided in several studies across several student samples (e.g., Conley et al., 2004; Tsai et al., 2011; Chen, 2012). For an overview of these studies, we kindly refer the reader to Kampa et al. (2016). Students were asked to indicate the extent to which they agreed with four statements, each of which represented the development and tentativeness of scientific knowledge (e.g., “New discoveries may imply that researchers in the future believe in ideas that are different from the ideas they believe in now.”) on a six-point agreement scale with extreme response categories anchored with a phrase (*1 = strongly disagree, 6 = strongly agree*). Scale reliabilities were acceptable, α = 0.90, ω = 0.87^3^. Please find the item formulations in **Table 1**.

**Table 1:** Item wordings, descriptive statistics, medians, skewnesses, and kurtosis of the epistemological beliefs and adaptability items.

Item	*M* (*SD*)	*Mdn*	Skewness	Kurtosis
**Epistemological beliefs – Development of scientific knowledge**				
**DE1.** Scientists now believe in ideas that are different from the ideas they used to believe in.	3.28 (1.34)	3	–0.36	–0.64
**DE2.** Some of the ideas stated in science books might be changed in the future.	3.82 (1.24)	4	–0.87	–0.06
**DE3.** There are some scientific problems that even scientists cannot solve.	3.84 (1.32)	4	–0.90	–0.19
**DE4.** New discoveries may imply that researchers in the future believe in ideas that are different from the ideas they believe in now.	3.81 (1.26)	4	–0.81	–0.24
**Adaptability – Cognitive-behavioral aspect**				
In a new and unfamiliar situation, …				
**CB1.** I am able to come up with and evaluate a number of possible options for solutions.	2.84 (1.21)	3	–0.16	–0.34
**CB2.** I am able to revise the way I reason and think about the new situation.	2.76 (1.15)	3	–0.07	–0.40
**CB3.** I am able to adjust my thinking and my expectations.	3.09 (1.20)	3	–0.31	–0.35
**CB4.** I am able to reformulate a weakened hypothesis and make plans for collecting appropriate information to test my new hypothesis.	2.74 (1.25)	3	–0.07	–0.49
**CB5.** I am able to adjust and change the way I do things such as applying new strategies for solving problems.	3.32 (1.22)	3	–0.47	–0.30
**CB6.** I am able to reason on how a change in an experiment affects the result.	2.97 (1.22)	3	–0.20	–0.42
**CB7.** I am able to identify arguments for and against a possible problem solution.	3.17 (1.23)	3	–0.34	–0.40
**CB8.** I am able to think of different possible problem solutions.	3.04 (1.19)	3	–0.26	–0.35
**Adaptability – Affective-emotional aspect**				
In a new and unfamiliar situation, …				
**AE1.** I am able to reduce my fear of failing.	2.83 (1.41)	3	–0.14	–0.75
**AE2.** I am able to reduce my frustration and irritation.	2.71 (1.37)	3	–0.10	–0.73
**AE3.** I am able to draw on my positive thoughts and emotions in order to succeed, such as enjoyment and satisfaction.	3.09 (1.31)	3	–0.36	–0.50
**AE4.** I am able to draw on my expectations of success.	3.01 (1.32)	3	–0.31	–0.55
**AE5.** I am able to draw on my expectations that I can certainly master challenges.	3.04 (1.33)	3	–0.32	–0.56

#### Perceived Adaptability

As noted earlier on, adaptability refers to a person’s capacity to adjust his or her thinking, behavior, affect, and emotions to novel, uncertain, or changing situations (VandenBos, 2007; Martin et al., 2012b). This capacity can be measured in many ways, be it with the help of performance-based assessments that present students with novel, uncertain, or dynamic problem situations (Scherer, 2015) or self-report scales that reflect on students’ perceptions of their adaptability (Martin et al., 2013). In the current study, we adopted Martin et al.’s (2013) perceived adaptability scale, because it provides an economical and valid measure of the construct (see also Martin et al., 2012b). Moreover, this scale distinguishes between two subscales, cognitive-behavioral and affective-emotional adaptability. The English item wordings are shown in **Table 1**. Students were asked to indicate the degree to which they agreed with statements that represented cognitive-behavioral (eight items) and affective adaptability (five items) on a six-point agreement scale with extreme response categories anchored with a phrase (*1 = strongly disagree, 6 = strongly agree*). Both subscales showed acceptable reliabilities (cognitive-behavioral: α = 0.93, ω = 0.84, affective-emotional: α = 0.88, ω = 0.77).

#### Immigration Status

Students’ immigration status was coded as 0 (*native Norwegian*) and 1 (*first- or second-generation immigration background*).

### Statistical Analyses

To examine the relation between students’ perceived adaptability and their EBDE with or without taking into account their immigration background (RQ1a and 1b), we established latent variable models with correlated factors. To further examine the extent to which EBDE and adaptability were related on a measurement level, we performed moderated non-linear factor analysis (RQ2a and 2b), which unites the features of multi-group and multiple-indicators-multiple-causes approaches to measurement invariance and DIF (Bauer, 2016).

#### Measurement Invariance and Differential Item Functioning (DIF)

Generally speaking, the concept of measurement invariance forms a prerequisite for performing valid comparisons of a measure across groups of items or persons (Millsap, 2011). A more formal definition of the concept entails “a situation in which a scale or construct provides the same results across several different samples or populations” (American Psychological Association [APA], 2014, p. 211). This definition implies that the properties of the measure and the corresponding measurement model are comparable across the samples or populations of interest. This characteristic is of particular importance, because in cases where a measure functions differently (i.e., its properties vary across samples or populations), “analyses of individual differences may not reflect the phenomena of interest” (Bauer, 2016, p. 1). As a consequence, if measurement invariance cannot be established, the probabilities of two individuals of the same ability or trait level in responding to the measure in a particular way are not equal, that is, DIF occurs (Millsap, 2011). Testing for measurement invariance or any deviation from it is of relevance for the present study, because it focuses on the extent to which the functioning of the EBDE scale is influenced by students’ adaptability (RQ2a), immigration status, or their interactions (RQ2b).

#### Moderated Non-linear Factor Analysis (MNLFA)

Since we were interested in the extent to which the measurement of students’ EBDE was invariant across the *continuum* of perceived adaptability, multi-group confirmatory factor analysis – an approach that is commonly applied to examine measurement invariance across *categorical* grouping variables – could not be applied. Multi-group confirmatory factor analysis is based on the assumption that the grouping variable is categorical and entails clearly defined levels or group memberships for each student in the sample (e.g., gender, level of socio-economic status); yet, in many applications, the “grouping” variable of interest is often continuous (e.g., age, motivation; Tucker-Drob, 2009; Curran et al., 2014). In order to circumvent any antagonisms in terminology, we refer a continuous “grouping” variable to as a covariate that might interact with parameters in measurement models. Hildebrandt et al. (2016) established an alternative term, namely “continuous context variable”. Although categorizing continuous context variables such as age or, in our case, perceived adaptability may provide a solution to this problem, there is a growing body of evidence that this approach is problematic, mainly because information on individual differences within groups gets lost (MacCallum et al., 2002; Hildebrandt et al., 2009). As a consequence, Hildebrandt et al. (2016, p. 258) suggested that continuous context variables “should be treated as continuous variables, not as categorical variables.” We therefore decided to follow Hildebrandt et al.’s (2016) suggestion and applied statistical models that treated the grouping variable as a continuous variable.

Among the existing continuous approaches to measurement invariance (for a brief overview, we refer the reader to Hildebrandt et al., 2016), moderated non-linear factor analysis (MNLFA) has been put forward as a most flexible (Curran et al., 2014). In fact, MNLFA allows researchers to not only examine measurement invariance along a continuum of a covariate but also across categorical variables and interactions between different two types of variables at the same time. All model parameters (i.e., factor loadings, intercepts, residual variables, factor means, variances, and covariances) can vary as a function of covariates that show differences between individuals (Bauer, 2016). These possibilities make MNLFA a rather flexible approach to measurement invariance (Bauer and Hussong, 2009).

Moderated non-linear factor analysis is based on the idea of model parameter moderation with respect to individual characteristics (Bauer, 2016). In our case, we study this moderation along the continuum of perceived adaptability and, later on, across its interaction with immigration status. In a factor model, *Y_im_ = v_i_(m) + λ_i_(m) . η_m_ + 𝜀_im_*, where Y_i_ denotes the students’ responses to item i, v_i_ the item intercepts, λ_i_ the item factor loadings, η the latent variable, and 𝜀_i_ the residual variances, model parameters vary as a function of the moderating context variable m. Specifically, the factor loadings λ_i_ can be predicted by a function of m, for instance, *λ_i_(m) = λ_i0_ + λ_i1_ ⋅ m + u_λim_* with normally distributed and uncorrelated residuals u_λim_ (Hildebrandt et al., 2016). The same reasoning applies to the item intercepts v_i_ and the factor means. For the factor variances, covariances, and item residuals, linear functions for m may lead to negative variances or correlations above 1 (“Heywood cases”; Dillon et al., 1987). These cases may cause severe estimation problems and consequently compromise the trustworthiness of estimated model parameters. As a consequence, Bauer (2016) argued that log-linear functions for m are more appropriate to study the moderation of factor or item residual variances. For the moderation functions of covariances, we kindly refer the reader to Bauer (2016) who proposed a suitable transformation of covariances.

In order to address research questions 2a and 2b on the measurement invariance of the EB scales along the continuum of perceived adaptability, we conduct MNLFA and examine the extent to which critical parameters in the EB measurement model (i.e., factor means and variances, item factor loadings and intercepts) are moderated by perceived adaptability, immigration status, and their interaction.

#### Estimator, Clustered Sample Structure, and Missing Data

All analyses were conducted in the statistical package M*plus* 7.3 (Muthén and Muthén, 1998–2015), with robust maximum likelihood (MLR) estimation with categorically treated item responses. The MLR estimation approach provides standard errors that are robust against non-normality of item responses when at least five response categories are used (e.g., Rhemtulla et al., 2012; Marsh et al., 2013; Li, 2016). The resultant measurement models represent Graded Response Models (GRMs), in which item factor loadings in a GRM can be interpreted comparable to those in the two-parametric logistic model (GRMs; Samejima, 1968). Item “intercepts” are based on log odds and represent the probability of moving from a given response category to the any higher category (Brown and Maydeu-Olivares, 2012). Despite the categorical treatment of item responses in this study, we will use the term “intercept” in the remainder of this paper. Potential covariate effects on item intercepts are comparable to those in ordinal logistic regression (Elosua and Wells, 2013). In both the measurement and the MNLFA models, the clustering of student data in schools was taken into account by a correction of the standard errors of model parameters (M*Plus* option TYPE = COMPLEX; Satorra and Bentler, 2010). Missing data were handled with the help of the full-information-maximum-likelihood (FIML) procedure under the assumption that they occurred randomly (Forero and Maydeu-Olivares, 2009; Enders, 2010). In the current data, less than 8.1% of item responses were missing.

## Results

To address our research questions, we first ensured that the measurement models of perceived adaptability and epistemological beliefs showed sufficient psychometric properties. This examination was then followed by the performance of structural equation modeling to estimate the correlations among the two adaptability dimensions, EBDE, and immigration background as a potential covariate (RQs 1a and 1b). Finally, we conducted moderated factor analysis to test whether the EBDE measure was invariant with respect to adaptability—this analysis included immigration status as another, possible covariate (RQs 2a and 2b).

### Descriptive Statistics and Measurement Models

Before examining the relations between perceived adaptability and EBDE at both the construct and the measurement level, we evaluated the descriptive statistics of items along with additional descriptors of their distributions. **Table 1** shows the resultant statistics. There was a tendency toward more sophisticated rather than naïve epistemological beliefs, whereas no clear tendencies could be observed for the adaptability scales. Regarding the shapes of item response distributions, epistemological beliefs items showed higher absolute skewness and kurtosis, confirming the aforementioned tendency. Although robust estimation procedures that treat item responses continuously are able to account for non-normal item response distributions (Rhemtulla et al., 2012), we treated item responses categorically to rule out potential bias in factor models that contain interaction effects.

In order to examine both the relations among perceived adaptability and EBDE along with the invariance of the epistemological beliefs measure, appropriate measurement models of the constructs must be established in a first step (Bauer, 2016). For students’ EBDE, items showed sufficiently high factor loadings (standardized λ = 0.78–0.92). For the adaptability scale, Martin et al. (2012b) proposed a two-dimensional factor structure that distinguishes between a cognitive-behavioral and an affective-emotional component of adaptability. Indeed, a two-dimensional measurement model (LL = -27324.2, Npar = 79, SCF = 1.1531; AIC = 54842.4, BIC = 55268.0, aBIC = 55017.0) performed significantly better in terms of model fit than a unidimensional model (LL = -28133.5, Npar = 78, SCF = 1.1525; AIC = 56423.1, BIC = 56843.3, aBIC = 56595.5), as indicated by the Satorra–Bentler corrected likelihood ratio test, Δ(-2LL)[1] = 1348.9, *p* < 0.01. Factor loadings were sufficiently high for both dimensions (cognitive-behavioral: standardized λ = 0.72–0.84; affective-emotional: standardized λ = 0.65–0.86). The resultant correlation between cognitive-behavioral and affective-emotional adaptability was substantial, ρ = 0.68, *p* < 0.01. Given the complexity of the MNLFA models that will be further specified, the computationally demanding procedures to estimate latent variable models with categorically treated item responses (Bauer, 2016) and the strong correlation between the two adaptability components that might cause multicollinearity in models with interactions, we decided to take a “divide and conquer” approach in the pursuit to quantify the relations between perceived adaptability and EBDE at the measurement level. Consequently, the MNLFA models were specified for cognitive-behavioral and affective-emotional adaptability separately.

### Correlations Between Perceived Adaptability and EBDE (RQ1a and 1b)

Addressing our first research question (RQ1a), we identified positive and significant correlations between EBDE and cognitive-behavioral adaptability (ρ = 0.56, *p* < 0.01), and EBDE and affective-emotional adaptability (ρ = 0.39, *p* < 0.01). Extending the perspective on these correlations by adding an interaction term between adaptability and immigration status revealed only significant main effects of adaptability and immigration status. More specifically, in a model comprising cognitive-behavioral adaptability, immigration status, and their interaction – defined with the help of the XWITH option in M*plus* (Muthén and Muthén, 1998–2015) – revealed significant main effects for adaptability (*B* = 0.47, *SE* = 0.04, *p* < 0.001) and immigration status (*B* = -0.31, *SE* = 0.15, *p* < 0.05) on EBDE, yet no significant interaction effect (*B* = -0.0, *SE* = 0.08, *p* = 0.59). In the corresponding model for affective-emotional adaptability, only the main effect of affective-emotional adaptability existed (*B* = 0.48, *SE* = 0.06, *p* < 0.001); the effects of immigration status (*B* = -0.25, *SE* = 0.17, *p* = 0.09) and its interaction with adaptability (*B* = -0.01, *SE* = 0.11, *p* = 0.96) were insignificant. We further note that there were no effects of immigration status on neither cognitive-behavioral (*d* = -0.04, *p* = 0.76) nor affective-emotional adaptability (*d* = -0.06, *p* = 0.49). In response to RQ1b, we argue that there is no evidence that the relations between adaptability and EBDE are subject to differences between native Norwegian students and students with immigration background.

### Moderated Non-linear Factor Analysis (RQ2a and 2b)

Addressing our second research question, which was concerned with the extent to which the measurement of EBDE was subject to DIF due to (a) individual differences in adaptability and (b) interaction effects between adaptability and immigration status, we performed MNLFA, treating adaptability and immigration status as covariates and allowing for possible non-linear effects.

#### Covariate Effects of Adaptability

As a first step to approach RQ2a, we examined whether single EBDE items exhibited DIF, using the grand-mean centered adaptability sum scores as covariates. These models assumed linear and quadratic covariate effects on the EBDE factor mean, variance, item factor loadings, and intercepts, and they allowed us to flag DIF items. The detailed results of these analyses are presented in the Supplementary Appendices A, B. For both cognitive-behavioral and affective-emotional adaptability, only items DE1 and DE2 showed linear and quadratic DIF effects on intercepts. Factor means could be predicted by adaptability; the factor variance could also be predicted by adaptability in a quadratic model. A sample M*plus* code for specifying the underlying model is presented in the Supplementary Appendix C.

**FIGURE 2:**
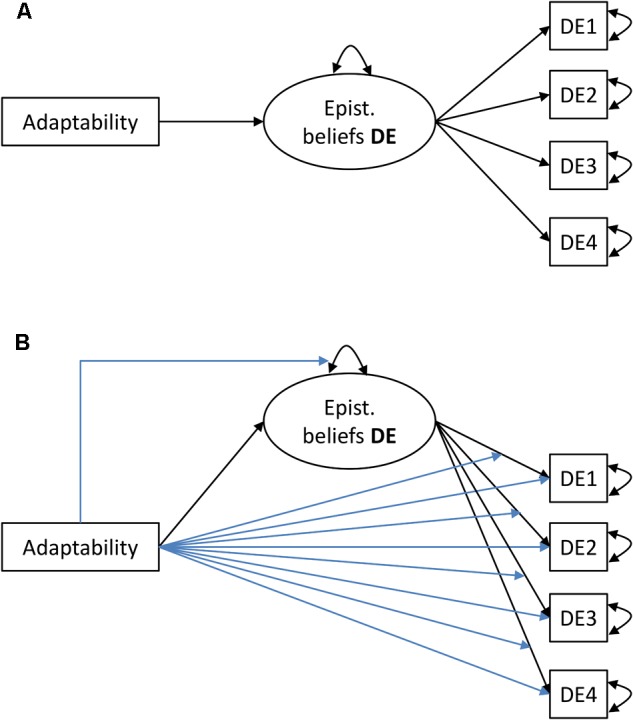
**(A)** Baseline model with adaptability as covariate that predicts the factor mean; **(B)** MNLFA model with adaptability as covariate that predicts the factor mean and variance as well as the item loadings and intercepts. DE, Epistemological beliefs in the development of scientific knowledge.

As a second step, we collated the effects that were identified in the first step; the resultant models are shown in **Table 2**. For both adaptability dimensions, the linear effects on the factor mean and the quadratic effects on the factor variance sustained. Hence, higher adaptability or, more precisely, larger positive deviations from the grand mean of adaptability were associated with more sophisticated EBDE. Variability in EBDE decreased along the adaptability continuum first and increased later, as suggested by the quadratic effect. At the item level, items DE1 and DE2 exhibited intercept DIF with linear covariate effects. These effects differed between the two items such that higher adaptability was associated with a higher probability of moving from one response category to any higher category on the EBDE logit scale for item DE1; the opposite relation was apparent for item DE2. Covariate effects on factor loadings did not exist. To strengthen the evidence for the existence of item DIF effects, we compared the MNLFA model to a baseline model which did not contain any DIF effects (see **Figure 2**). For both dimensions of adaptability, the difference in model fit between the baseline and the DIF effects models was statistically significant (see **Table 2**). This finding strengthened the empirical preference of the MNLFA models with DIF effects.

**Table 2:** Unstandardized parameter estimates from the MNLFA models with adaptability as covariate.

	Cognitive-behavioral adaptability				Affective-emotional adaptability			
Parameter	Baseline model without DIF effects		MNLFA with DIF effects		Baseline model without DIF effects		MNLFA with DIF effects	
	CB	CB^2^	CB	CB^2^	AE	AE^2^	AE	AE^2^
**Covariate effects**								
DE factor								
Mean	0.77 (0.05)^∗∗^	0.03 (0.03)	0.76 (0.05)^∗∗^	–	0.41 (0.04)^∗∗^	0.05 (0.03)^#^	0.41 (0.04)^∗∗^	0.05 (0.03)^#^
Variance^a^	–0.17 (0.06)^∗∗^	0.19 (0.04)^∗∗^	–0.20 (0.06)^∗∗^	0.17 (0.03)^∗∗^	–0.20 (0.05)^∗∗^	0.16 (0.03)^∗∗^	–0.22 (0.05)^∗∗^	0.15 (0.03)^∗∗^
Item DE1								
Loading	–	–	–	–	–	–	–	–
Intercept	–	–	0.25 (0.08)^∗∗^	0.09 (0.05)^#^	–	–	0.17 (0.06)^∗∗^	–
Item DE2								
Loading	–	–	–	–	–	–	–	–
Intercept	–	–	–0.21 (0.10)^∗^	–	–	–	–0.18 (0.09)^∗^	–
Item DE3								
Loading	–	–	–	–	–	–	–	–
Intercept	–	–	–	–	–	–	–	–
Item DE4								
Loading	–	–	–	–	–	–	–	–
Intercept	–	–	–	–	–	–	–	–
**Model fit information**								
LL	–7483.7		–7472.7		–7598.7		–7589.3	
NPar	28		30		28		30	
SCF	1.2210		1.2248		1.2105		1.2033	
AIC	15023.4		15005.4		15253.3		15238.6	
BIC	15173.8		15166.5		15403.7		15399.7	
aBIC	15084.8		15071.2		15314.7		15304.4	
**Model comparisons^b^**								
Δ(-2LL) (ΔNpar)	17.2 (2)^∗∗^				17.1 (2)^∗∗^			

*DIF, Differential item functioning; CB, Cognitive-behavioral adaptability score; AE, Affective-emotional adaptability score; DE, Epistemological beliefs in the development of scientific knowledge; LL, Loglikelihood value; Npar, Number of free parameters; SCF, Scaling correction factor; aBIC, Sample-size adjusted BIC. ^*a*^A log-linear model was specified for the factor variance, variance = exp(*covariate*). ^*b*^Model comparisons were conducted using the Satorra–Bentler corrected Likelihood-ratio test (Satorra and Bentler, 2010). Standard errors are shown in brackets. ^#^*p* < 0.10, ^∗^*p* < 0.05, ^∗∗^*p* < 0.01.*

#### Covariate Effects of Immigration Status, Adaptability, and Their Interaction

Following the same approach taken to address RQ2a, we first examined whether there existed covariate effects on factor means, variance, and item parameters across students’ immigration status to address RQ2b. The results of this stepwise procedure are detailed in the Supplementary Appendix D. Students’ immigration status showed only significant effects on the factor mean and the intercept of item DE1. The effect on the factor mean was negative; this indicates that native Norwegian students had more sophisticated EBDE than students with an immigration background. At the same time, students with immigration status had a higher probability of moving from one response category to any higher category in item DE1. Collating these effects resulted in a full model that fitted the data significantly better than the baseline model (see **Table 3**). Consequently, the measurement of EBDE was not completely comparable between native Norwegian students and students with an immigration background.

**Table 3:** Unstandardized parameter estimates from the MNLFA models with immigration status as covariate.

Parameters	Baseline model without item DIF	Model with item DIF
**Covariate effects**		
DE factor		
Mean	–0.17 (0.09)^∗^	–0.33 (0.15)^∗^
Variance^a^	–0.15 (0.15)	–
Item DE1		
Loading	–	–
Intercept	–	0.35 (0.15)^∗^
Item DE2		
Loading	–	–
Intercept	–	–
Item DE3		
Loading	–	–
Intercept	–	–
Item DE4		
Loading	–	–
Intercept	–	–
**Model fit information**		
LL	–7601.9	–7600.1
Npar	26	27
SCF	1.2228	1.1659
AIC	15255.8	15254.1
BIC	15395.0	15398.7
aBIC	15312.4	15312.9
**Model comparison**		
Δ(-2LL) (ΔNpar)	11.5 (1)^∗∗^	

*DIF, Differential item functioning; DE, Epistemological beliefs in the development of scientific knowledge; LL, Loglikelihood value; Npar, Number of free parameters; SCF, Scaling correction factor; aBIC, Sample-size adjusted BIC. ^*a*^A log-linear model was specified for the factor variance, variance = exp(*covariate*). ^*b*^Model comparisons were conducted using the Satorra–Bentler corrected Likelihood-ratio test (Satorra and Bentler, 2010). Standard errors are shown in brackets. ^#^*p* < 0.10, ^∗^*p* < 0.05, ^∗∗^*p* < 0.01.*

**FIGURE 3:**
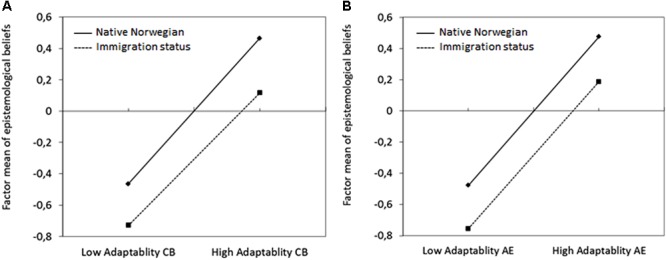
Interaction effects between students’ immigration status and **(A)** cognitive-behavioral adaptability (CB) and **(B)** affective-emotional adaptability (AE) on the factor mean of epistemological beliefs in the development of scientific knowledge.

**FIGURE 4:**
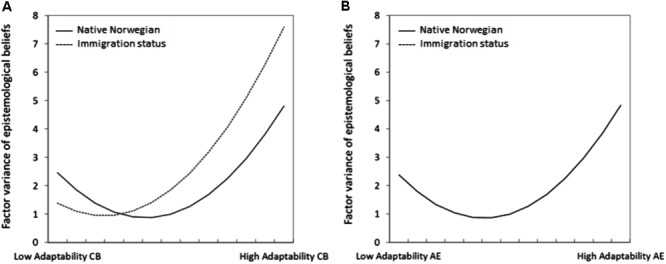
Interaction effects between students’ immigration status and **(A)** cognitive-behavioral adaptability (CB) and **(B)** affective-emotional adaptability (AE) on the factor variance of epistemological beliefs in the development of scientific knowledge.

Finally, we examined the covariates effects of adaptability, immigration status, and their interactions including the corresponding quadratic terms. Once again, we performed a stepwise procedure to disentangle the significance of effects (see Supplementary Appendices E,F). These effects were again collated into a full model for each of the two adaptability dimensions (see **Tables 4, 5**). For cognitive-behavioral adaptability, linear effects on the factor mean and quadratic effects for the factor variance existed. Moreover, the significant effects on item intercepts sustained for items DE1 and DE2. In addition to these effects, a positive interaction effect between adaptability and immigration status on the factor variance of EBDE could be identified, whereas the interaction effects did not exist neither for the factor mean nor the item parameters. **Figure 3A** depicts the effects on the factor mean. Clearly, the relations between adaptability and the EBDE factor mean did not differ significantly between native Norwegian students and students with immigration background. In contrast, the effects on the factor variance were moderated by immigration status, as shown in **Figure 4A**. Hence, variation in EBDE reached a global minimum at lower adaptability scores for students with an immigration background, and moderate levels for native Norwegian students. The underlying MNLFA model fitted the data significantly better than the baseline model, Δ(-2LL)[7] = 391.5, *p* < 0.01. For affective-emotional adaptability, the linear effects on the factor mean and the quadratic effects on the factor variance could be replicated. Yet, only the intercept effect on item DE1 existed; interaction effects could not be identified. **Figure 3B** visualizes the factor mean differences across student with and without immigration status; **Figure 4B** shows the quadratic variance effects, which were not moderated by immigration status. The underlying MNLFA model for affective-emotional adaptability fitted the data significantly better than the baseline model, Δ(-2LL)[6] = 229.1, *p* < 0.01.

**Table 4:** Unstandardized parameter estimates from the MNLFA models with cognitive-behavioral adaptability, students’ immigration status, and their interaction as covariates.

	*MNLFA assuming item DIF*					

Parameters	CB	IMMIG	CB^2^	IMMIG × CB	IMMIG × CB^2^
**Covariate effects**					
DE factor					
Mean	0.78 (0.06)^∗∗^	–	–	–	–
Variance^a^	–0.24 (0.06)^∗∗^	–0.16 (0.14)	0.16 (0.04)^∗∗^	0.25 (0.11)^∗^	–
Item DE1					
Loading	–	–	–	–	–
Intercept	0.22 (0.07)^∗∗^	0.30 (0.15)^∗^	0.07 (0.05)	–	–
Item DE2					
Loading	–	–	–	–	–
Intercept	–0.25 (0.11)^∗^	–	–	–	–
Item DE3					
Loading	–	–	–	–	–
Intercept	–0.14 (0.09)	–	–	0.25 (0.22)	–
Item DE4					
Loading	–	–	–	–	–
Intercept	–	–	–	–	–
**Model fit information**					
LL	–7294.8				
Npar	35				
SCF	1.1698				
AIC	14659.7				
BIC	14846.9				
aBIC	14735.7				

*DIF, Differential item functioning; CB, Cognitive-behavioral adaptability score; IMMIG, Immigration status (*1 = Immigration status, 0 = Native Norwegian*); DE, Epistemological beliefs in the development of scientific knowledge; LL, Loglikelihood value; Npar, Number of free parameters; SCF, Scaling correction factor; aBIC, Sample-size adjusted BIC. ^a^A log-linear model was specified for the factor variance, variance = exp(*covariate*). Standard errors are shown in brackets. ^#^*p* < 0.10, ^∗^*p* < 0.05, ^∗∗^*p* < 0.01.*

**Table 5:** Unstandardized parameter estimates from the MNLFA models with **affective-emotional adaptability**, students’ immigration status, and their interaction as covariates.

	*MNLFA assuming item DIF*					

Parameters	AE	IMMIG	AE^2^	IMMIG × AE	IMMIG × AE^2^
**Covariate effects**					
DE factor					
Mean	0.41 (0.04)^∗∗^	–	0.04 (0.03)	–	–
Variance^a^	–0.20 (0.05)^∗∗^	–	0.15 (0.03)^∗∗^	–	–
Item DE1					
Loading	–	–	–	–	–
Intercept	0.17 (0.06)^∗∗^	–	–	–	–
Item DE2					
Loading	–	–	–	–	–
Intercept	–0.17 (0.09)^#^	–	–	–	–
Item DE3					
Loading	–	–	–	–	–
Intercept	–	–	–	0.25 (0.22)	–
Item DE4					
Loading	–	–	–	–	–
Intercept	–	–	–	–0.10 (0.23)	–
**Model fit information**					
LL	–7865.7				
Npar	34				
SCF	1.4083				
AIC	15799.3				
BIC	15981.4				
aBIC	15873.4				

*DIF, Differential item functioning; AE, Affective-emotional adaptability score; IMMIG, Immigration status (*1 = Immigration status, 0 = Native Norwegian*); DE, Epistemological beliefs in the development of scientific knowledge; LL, Loglikelihood value; Npar, Number of free parameters; SCF, Scaling correction factor; aBIC, Sample-size adjusted BIC. ^a^A log-linear model was specified for the factor variance, variance = exp(*covariate*). Standard errors are shown in brackets. ^#^*p* < 0.10, ^∗^*p* < 0.05, ^∗∗^*p* < 0.01.*

Overall, our response to RQ2b is this: Given that item DIF effects for adaptability and immigration status existed, full measurement invariance could not be established. At the same time, the interaction between adaptability and immigration status did not cause any DIF; in contrast, the factor variance was affected by the interaction for cognitive-behavioral adaptability.

## Discussion

The present study put to test the assumption that epistemological beliefs in the development of scientific knowledge and cognitive flexibility are correlated. Focusing on adaptability as a broader concept that represents both cognitive-behavioral and affective-emotional flexibility, we identified a positive and significant correlation. Albeit we confirmed the hypothesized correlation at the construct level, we further found that individual differences in students’ perceived adaptability were associated with differences in the factor mean, variance, and selected item parameters in the EBDE measurement model. This finding suggested the differential functioning of the EBDE measure as a function of adaptability because some item intercepts and factor variances—essential parameters of the measurement model—depended on the adaptability score. Similarly, effects of immigration status occurred, although interaction effects with adaptability were not severe. Overall, there was convincing evidence that students’ perceptions of their adaptability were related to their beliefs about the development and tentativeness of scientific knowledge.

### Relations Among Perceived Adaptability and EBDE at the Construct Level

The results of this study uncovered a positive and statistically significant correlation between the two components of perceived adaptability and students’ EBDE. This correlation clearly suggests a relation at the construct level and supports the hypothesized link between the two constructs (Stahl, 2011). One potential interpretation of this link is based on the assumption that both constructs point to rather similar concepts, that is, the changing nature of scientific knowledge and individual’s approaches to dealing with these changes (Conley et al., 2004; Martin et al., 2013). Even though perceived adaptability and EBDE clearly represent concepts that tap different belief systems, this common denominator might have caused the significant relation between them. Even further, from a measurement perspective, the construct measures are well-aligned – especially with respect to the concepts they refer to in specific items; for instance, both refer to changes in scientific knowledge that are due to scientific progress and experimentation. Overall, the evidence presented on the link between EBDE and adaptability supports Stahl’s (2011) hypothesis.

Moreover, this evidence extends Stahl’s (2011) argumentation in that the Adaptability—EBDE relation does not only exist for the cognitive-behavioral component of adaptability, the component that Stahl (2011) focused on his hypothesis, but also for the affective-emotional component. In fact, the positive relation between the affective-emotional aspect and EBDE indicates that the epistemological beliefs system is not only connected to cognition and behavior. This finding further emphasizes that views about the world might include emotional components (Dennis et al., 2013; Gill and Hardin, 2015). Yet, to strengthen this argument and perspective, an extended EBDE assessment is needed that comprises cognitive-behavioral as well as affective-emotional components – just as the adaptability assessment does. Finally, although affective-emotional adaptability and EBDE were correlated, the relation was weaker as compared to cognitive-behavioral adaptability. This finding, once again, emphasizes that EBDE clearly tap a beliefs system that is closely related to cognition and metacognition, whereas affect and emotions may play a lesser role (Hofer and Pintrich, 1997).

From a more beliefs-oriented perspective, the identified correlation between perceived adaptability and EBDE suggests a connection between two different beliefs systems – that of epistemological beliefs (i.e., beliefs about external objects, contents, or procedures) with that of self-beliefs (i.e., perceptions about the openness and capacity to adjust in novel, uncertain, and changing situations). This finding is in line with what Schoenfeld (1983) proposed when he distinguished between four key beliefs systems in the context of mathematics learning (i.e., beliefs about the self, the nature of mathematics, the environment, and the topic) – these beliefs are considered distinct, yet connected. It also extends the current body of research in that it provides a link between adaptability and *epistemological* beliefs rather than adaptability and *efforts-related beliefs* (e.g., implicit theories; Martin et al., 2013). Interestingly, Op’t Eynde and De Corte (2003) found a similar distinction between EBDE and self-beliefs in mathematics that clearly indicated the distinction between the two types of beliefs. Other researchers supported this distinction for further domains and self-beliefs (Franklin-Guy, 2006; Ricco et al., 2010; Kampa et al., 2016). Overall, EBDE and self-beliefs are intertwined (Hofer and Pintrich, 1997) – it therefore seems as if beliefs about science are to some extent projected through the lens of the self.

Adding a between-person differences perspective to the Adaptability—EBDE relation, we neither identified effects of immigration status nor interaction effects between immigration status and adaptability. The hypothesized differences in adaptability between native students and students with an immigration background be identified; furthermore, they did not translate into differences in the relation to EBDE – as a matter of fact, the correlations were unaffected. Although our study can by no means deliver a causal explanation for this finding, it seems as if students’ self-perceptions translate into their beliefs about the development of scientific knowledge independent of their prior exposure to different cultures, languages, and background in the context of immigration. We believe that in-depth studies on individual trajectories of both perceived adaptability and EBDE might help explain this finding.

From an educational perspective, both adaptability and sophisticated epistemological beliefs are considered critical dispositions that help students to become reflective citizens in ever-changing and information-lean societies (Pulakos et al., 2002; Liem and Martin, 2015). In fact, both contribute to the development of students’ critical thinking and problem solving skills (Abrami et al., 2008; Heitmann et al., 2014), and can be considered educational outcomes and learning goals at the same time (Ricco et al., 2010; Green and Hood, 2013; Kampa et al., 2016). The current study emphasizes this importance as it shows that perceived adaptability and EBDE are connected. Moreover, this relation stresses the possible dependence between the two constructs in a sense that fostering the one might also translate into gains of the other. Educators should therefore not forget to consider self-beliefs when aiming at enhancing students’ EBDE. This claim, of course, needs further attention, and we would like to encourage experimental studies that put it to test. These studies may also explore the role and relevance of social cognition and the theory of mind—two possible factors that could provide additional insights into the link between several belief systems.

### Relations Among Perceived Adaptability and EBDE at the Measurement Level

As mentioned earlier on, we examined the Adaptability—EBDE relation not only at the construct level but also at the measurement level. In this sense, we attempted to supplement the correlational findings by evidence for the equivalent functioning of the EBDE measure across different adaptability scores. Moving from the construct level to the very bits and pieces that form the construct of EBDE – that is, the parameters in its measurement model – addresses issues of validity of the resultant EBDE scores (American Educational Research Association [AERA], 2014). In fact, examining potential different item functioning across groups of students or along a continuous variable is considered essential in the creation of a validity argument (Pellegrino et al., 2016).

To this end, the findings of our study suggested that some parameters in the EBDE measurement model were moderated by students’ perceived adaptability and immigration status. Considering these moderation effects, we have evidence that EBDE and adaptability are not only connected at the construct level – instead, individual differences in adaptability determine how the EBDE functions. Once again, we believe that this result suggests that students might perceive the development of scientific knowledge through the lenses of their self-perception (Kampa et al., 2016). Even further, students’ responses to some of the EBDE items, the resultant variation in the EBDE factor and its mean depend on their perceived adaptability. Consequently, we argue that in order for researchers to make sense of item responses on EBDE measures, these measures should be accompanied with some measures of students’ self-beliefs; the latter may help explain variation in EBDE at both the construct and the item level. At the same time, we notice that the present study cannot deliver evidence on the causality of effects. More precisely, it might well be that students’ EBDE further influence the measurement of their perceived adaptability. Nonetheless, the assumption that self-beliefs are the lenses through which we perceive the world is well-supported in other studies (Hofer and Pintrich, 1997; Kampa et al., 2016).

Furthermore, the DIF with respect to immigration status also calls into question the comparability of EBDE scores. Potential reasons for this finding are numerous and might, for instance, relate to subtleties in item formulations that might have caused a differential understanding of their meaning or the meaning of single terms, or differences in reading proficiency (Millsap, 2011; Huang et al., 2016; Syu and Zhang, 2017).

From a methodological perspective, the approach taken to address the DIF along the continuum of adaptability and across immigration status – moderated non-linear factor analysis – was flexible enough to examine the moderation of model parameters regardless whether the covariates were continuous, categorical, or combinations thereof (Bauer, 2016). In fact, the possibility to determine the degree to which DIF exists along a continuous covariate is intriguing because it no longer requires the categorization of covariates into arbitrary levels. This methodological advantage brings back the individual differences perspective to DIF testing (Curran et al., 2014). Apart from this, MNLFA goes beyond traditional measurement invariance testing as it allows for testing covariate effects not only on item factor loadings, intercepts, and residuals but also factor variances, means, and even covariances. Our study exemplified how MNLFA can be used to address a substantive research question that concerns the relation between two, educationally relevant constructs – perceived adaptability and EBDE.

Overall, we believe that this methodological perspective is critical because it brings to attention the validity of the EBDE scores from an individual differences perspective. In this respect, validity is not only a matter of measurement equivalence across clearly defined groups of students (e.g., native vs. immigrant students); it now becomes a matter of individual differences in a covariate. Together with Bauer (2016), we encourage researchers in the fields of assessment and measurement to consider adopting this perspective in order to better understand the processes and mechanisms behind students’ response behavior to items that tap EBDE or other beliefs. Moreover, we encourage researchers to further investigate the validity of the commonly used EBDE scale by examining its functioning in conjunction with other constructs (e.g., science self-beliefs, interest, implicit theories; Chen, 2012).

### Limitations

The present study has some limitations worth noting. First, the sample was restricted to Norwegian students. It is possible that the relation between perceived adaptability and EBDE – be it at the construct or measurement level – is subject to cultural and language differences. For instance, the existing body of research indicates that students’ EBDE vary across countries (Kampa et al., 2016). This variation may also appear in the functioning of the corresponding measures and items, therefore limiting their cross-country comparability. Consequently, our findings should be generalized with caution. Second, we employed self-report measures of both EBDE and adaptability. It is possible that the alternative measures – be it measures of EBDE derived from interviews or performance-based measures of adaptability – could reveal different relations between the two main constructs.

## Conclusion

This study has put to test the assumption that epistemological beliefs in the development of scientific knowledge and cognitive flexibility go together. Extending the perspective on ‘cognitive flexibility’ to a broader concept, adaptability, which also includes an affective-emotional aspect, we studied the hypothesized relation for a large sample of Norwegian students and found a positive and significant correlation. This correlation suggests that students’ views on their adaptability and the beliefs about the changing nature of scientific knowledge go together. At the same time, the results uncovered that the two constructs are related at the measurement level. More specifically, the measurement of EBDE was, to some extent, influenced by students’ perceived adaptability and their immigration status. This finding indicated that (a) the EBDE measure is subject to individual differences in adaptability and immigration status and that (b) the EBDE measure is not fully invariant with respect to these two covariates; the latter challenges the creation of a validity argument for the scores obtained from the EBDE measure and requires caution when interpreting these scores for individual students. Overall, it seems as if students perceive the world or, more precisely, the development of scientific knowledge therein, through the lenses of their own adaptability.

## Ethics Statement

This study was carried out in accordancewith the recommendations of the Individual Level Data Guidelines proposed by NSD with written informed consent from all subjects (in accordance with the Declaration of Helsinki). The study protocol was approved by the Norwegian Directorate of Education and Training (UDIR).

## Author Contributions

RS and ØG have initiated this study and developed/refined the measures. ØG was responsible for collecting and cleaning the data. RS conducted the analyses. RS drafted the manuscript and ØG contributed to its revision. Both authors engaged in the interpretation of the results.

## Conflict of Interest Statement

The authors declare that the research was conducted in the absence of any commercial or financial relationships that could be construed as a potential conflict of interest. The reviewer LF and handling Editor declared their shared affiliation.

## Footnotes

1 Uncertain situations, in this context, mostly refer to situations with unpredictable outcomes, in which some ongoing processes are not transparent (Osman, 2010).

2 English was the native language in the student samples Martin et al. (2012b) have studied.

3 Cronbach’s α and McDonald’s ω (specified as omega-hierarchical ω_h_) are reported as reliability coefficients for scales with ordinal responses (Revelle and Zinbarg, 2008; Trizano-Hermosilla and Alvarado, 2016). Both coefficients were calculated in the R package *psych* (Revelle, 2017). We note that they rely on slightly different assumptions on the underlying measurement model (Revelle and Zinbarg, 2008).
