# Supplementary material for: Observing the World Through Your Own Lenses – The Role of Perceived Adaptability for Epistemological Beliefs About the Development of Scientific Knowledge
Source: Front Psychol. 2018 Jun 20;9:1006. doi: 10.3389/fpsyg.2018.01006 (PMC6019493; doi:10.3389/fpsyg.2018.01006)
Supplement: Supplementary file 1 [file Data_Sheet_1.DOCX]

# Supplementary Material

Observing the World through Your Own Lenses – The Role of Perceived Adaptability for Epistemological Beliefs about the Development of Scientific Knowledge

Ronny Scherer

University of Oslo, Norway

Øystein Guttersrud

The Norwegian Centre for Science Education, Norway

**Contents**

| A) | Parameters of MNLFA Models with Cognitive-Behavioral Adaptability as Covariate | S2 |
| --- | --- | --- |
| B) | Parameters of MNLFA Models with Affective-Emotional Adaptability as Covariate | S4 |
| C) | Sample M*plus* code for the models assuming single-item DIF with adaptability as covariate (linear and quadratic effects; adopted from Bauer, 2016) | S6 |
| D) | Parameters of MNLFA Models with Immigration Status as Covariate | S8 |
| E) | Parameters of MNLFA Models with Cognitive-Behavioral Adaptability and Immigration Status as Covariates | S9 |
| F) | Parameters of MNLFA Models with Affective-Emotional Adaptability and Immigration Status as Covariates | S13 |
| G) | References | S17 |

## Parameters of MNLFA Models with Cognitive-Behavioral Adaptability as Covariate

*A1) Linear and quadratic effects models for items DE1 and DE2 (unstandardized)*

|  | *Models assuming single-item DIF* | | | | | |
| --- | --- | --- | --- | --- | --- | --- |
|  | Linear | Quadratic | | Linear | Quadratic | |
| *Covariate effects on parameters* | *CB* | *CB* | *CB^2^* | *CB* | *CB* | *CB^2^* |
| DE factor |  |  |  |  |  |  |
| Mean | 0.41 (0.03)** | 0.74 (0.05)** | 0.02 (0.03) | 0.44 (0.04)** | 0.80 (0.06)** | 0.02 (0.03) |
| Variance^a^ | -0.29 (0.04)** | -0.16 (0.06)** | 0.18 (0.04)** | -0.30 (0.04)** | -0.18 (0.06)** | 0.19 (0.04)** |
|  |  |  |  |  |  |  |
| Item DE1 |  |  |  |  |  |  |
| Factor loading | 0.00 (0.09) | -0.11 (0.07) | -0.02 (0.03) | – | – | – |
| Intercept | 0.26 (0.11)* | 0.31 (0.07)** | 0.12 (0.05)* | – | – | – |
|  |  |  |  |  |  |  |
| Item DE2 |  |  |  |  |  |  |
| Factor loading | – | – | – | 0.07 (0.11) | 0.02 (0.11) | -0.03 (0.05) |
| Intercept | – | – | – | -0.41 (0.15)** | -0.29 (0.11)** | -0.02 (0.09) |
|  |  |  |  |  |  |  |
| *Model fit information* |  |  |  |  |  |  |
| LL | -7488.4 | -7473.4 | | -7490.7 | -7478.5 | |
| Npar | 28 | 32 | | 28 | 32 | |
| SCF | 1.2505 | 1.1962 | | 1.2278 | 1.1902 | |
| AIC | 15032.8 | 15010.7 | | 15037.5 | 15020.9 | |
| BIC | 15183.2 | 15182.7 | | 15187.9 | 15192.8 | |
| aBIC | 15094.2 | 15081.0 | | 15099.0 | 15091.2 | |

*Note.* CB = Cognitive-behavioral adaptability score, DE = Epistemological beliefs in the development of scientific knowledge, LL = Loglikelihood value, Npar = Number of free parameters, SCF = Scaling correction factor, aBIC = Sample-size adjusted BIC. Statistically significant covariate effects are shaded in grey. ^a^ A log-linear model was specified for the factor variance, variance = exp(*covariate*). Standard errors are shown in brackets. * *p* < .05, ** *p* < .01

*A2) Linear and quadratic effects models for items DE3 and DE4 (unstandardized)*

|  | *Models assuming single-item DIF* | | | | | |
| --- | --- | --- | --- | --- | --- | --- |
|  | Linear | Quadratic | | Linear | Quadratic | |
| *Covariate effects on parameters* | *CB* | *CB* | *CB^2^* | *CB* | *CB* | *CB^2^* |
| DE factor |  |  |  |  |  |  |
| Mean | 0.23 (0.02)** | 0.78 (0.05)** | 0.03 (0.03) | 0.43 (0.04)** | 0.76 (0.05)** | 0.03 (0.03) |
| Variance^a^ | -0.28 (0.04)** | -0.17 (0.06)** | 0.19 (0.04)** | -0.28 (0.05)** | -0.17 (0.06)** | 0.18 (0.04)** |
|  |  |  |  |  |  |  |
| Item DE3 |  |  |  |  |  |  |
| Factor loading | -0.07 (0.09) | -0.02 (0.09) | -0.04 (0.04) | – | – | – |
| Intercept | 0.07 (0.08) | -0.09 (0.08) | -0.08 (0.06) | – | – | – |
|  |  |  |  |  |  |  |
| Item DE4 |  |  |  |  |  |  |
| Factor loading | – | – | – | -0.09 (0.12) | 0.02 (0.11) | 0.02 (0.08) |
| Intercept | – | – | – | 0.19 (0.17) | 0.05 (0.10) | -0.05 (0.07) |
|  |  |  |  |  |  |  |
| *Model fit information* |  |  |  |  |  |  |
| LL | -7611.3 | -7482.0 | | -7495.8 | -7482.9 | |
| Npar | 28 | 32 | | 28 | 32 | |
| SCF | 1.2025 | 1.1945 | | 1.2293 | 1.2137 | |
| AIC | 15278.7 | 15028.1 | | 15047.6 | 15029.8 | |
| BIC | 15429.1 | 15200.0 | | 15198.0 | 15201.7 | |
| aBIC | 15340.1 | 15098.3 | | 15109.0 | 15100.1 | |

*Note.* CB = Cognitive-behavioral adaptability score, DE = Epistemological beliefs in the development of scientific knowledge, LL = Loglikelihood value, Npar = Number of free parameters, SCF = Scaling correction factor, aBIC = Sample-size adjusted BIC. Statistically significant covariate effects are shaded in grey. ^a^A log-linear model was specified for the factor variance, variance = exp(*covariate*). Standard errors are shown in brackets. * *p* < .05, ** *p* < .01

## Parameters of MNLFA Models with Affective-Emotional Adaptability as Covariate

*B1) Linear and quadratic effects models for items DE1 and DE2 (unstandardized)*

|  | *Models assuming single-item DIF* | | | | | |
| --- | --- | --- | --- | --- | --- | --- |
|  | Linear | Quadratic | | Linear | Quadratic | |
| *Covariate effects on parameters* | *AE* | *AE* | *AE^2^* | *AE* | *AE* | *AE^2^* |
| DE factor |  |  |  |  |  |  |
| Mean | 0.22 (0.02)** | 0.39 (0.04)** | 0.05 (0.03)^#^ | 0.24 (0.02)** | 0.43 (0.04)** | 0.05 (0.03)^#^ |
| Variance^a^ | -0.29 (0.03)** | -0.20 (0.05)** | 0.15 (0.03)** | -0.29 (0.04)** | -0.21 (0.05)* | 0.15 (0.03)** |
|  |  |  |  |  |  |  |
| Item DE1 |  |  |  |  |  |  |
| Factor loading | -0.08 (0.07) | -0.06 (0.06) | -0.01 (0.04) | – | – | – |
| Intercept | 0.26 (0.06)** | 0.20 (0.06)** | -0.01 (0.05) | – | – | – |
|  |  |  |  |  |  |  |
| Item DE2 |  |  |  |  |  |  |
| Factor loading | – | – | – | -0.01 (0.13) | -0.01 (0.08) | -0.01 (0.04) |
| Intercept | – | – | – | -0.25 (0.10)* | -0.27 (0.09)** | -0.05 (0.05) |
|  |  |  |  |  |  |  |
| *Model fit information* |  |  |  |  |  |  |
| LL | -7603.8 | -7591.1 | | -7606.3 | -7593.0 | |
| Npar | 28 | 32 | | 32 | 32 | |
| SCF | 1.1989 | 1.1903 | | 1.2184 | 1.1693 | |
| AIC | 15263.6 | 15246.1 | | 15268.6 | 15250. | |
| BIC | 15413.9 | 15417.9 | | 15418.9 | 15421.9 | |
| aBIC | 15325.0 | 15316.3 | | 15330.0 | 15320.2 | |

*Note.* AE = Affective-emotional adaptability score, DE = Epistemological beliefs in the development of scientific knowledge, LL = Loglikelihood value, Npar = Number of free parameters, SCF = Scaling correction factor, aBIC = Sample-size adjusted BIC. Statistically significant covariate effects are shaded in grey. ^a^A log-linear model was specified for the factor variance, variance = exp(*covariate*). Standard errors are shown in brackets. ^#^ *p* < .10, * *p* < .05, ** *p* < .01

*B2) Linear and quadratic effects models for items DE3 and DE4 (unstandardized)*

|  | *Models assuming single-item DIF* | | | | | |
| --- | --- | --- | --- | --- | --- | --- |
|  | Linear | Quadratic | | Linear | Quadratic | |
| *Covariate effects on parameters* | *AE* | *AE* | *AE^2^* | *AE* | *AE* | *AE^2^* |
| DE factor |  |  |  |  |  |  |
| Mean | 0.23 (0.02)** | 0.40 (0.04)** | 0.05 (0.03)^#^ | 0.22 (0.02)** | 0.41 (0.04)** | 0.04 (0.03) |
| Variance^a^ | -0.28 (0.04)** | -0.20 (0.05)** | 0.15 (0.03)** | -0.31 (0.04)** | -0.21 (0.05)** | 0.16 (0.03)** |
|  |  |  |  |  |  |  |
| Item DE3 |  |  |  |  |  |  |
| Factor loading | -0.07 (0.09) | -0.03 (0.07) | 0.03 (0.04) | – | – | – |
| Intercept | 0.07 (0.08) | 0.02 (0.05) | 0.02 (0.05) | – | – | – |
|  |  |  |  |  |  |  |
| Item DE4 |  |  |  |  |  |  |
| Factor loading | – | – | – | 0.23 (0.16) | 0.09 (0.09) | -0.03 (0.06) |
| Intercept | – | – | – | -0.13 (0.13) | 0.05 (0.08) | 0.04 (0.04) |
|  |  |  |  |  |  |  |
| *Model fit information* |  |  |  |  |  |  |
| LL | -7611.3 | -7597.9 | | -7610.4 | 7596.6 | |
| Npar | 28 | 32 | | 28 | 32 | |
| SCF | 1.2025 | 1.1689 | | 1.2283 | 1.1901 | |
| AIC | 15278.7 | 15259.7 | | 15276.8 | 15257.3 | |
| BIC | 15429.1 | 15431.5 | | 15427.2 | 15429.1 | |
| aBIC | 15340.1 | 15329.9 | | 15338.2 | 15327.5 | |

*Note.* AE = Affective-emotional adaptability score, DE = Epistemological beliefs in the development of scientific knowledge, LL = Loglikelihood value, Npar = Number of free parameters, SCF = Scaling correction factor, aBIC = Sample-size adjusted BIC. Statistically significant covariate effects are shaded in grey. ^a^A log-linear model was specified for the factor variance, variance = exp(*covariate*). Standard errors are shown in brackets. ^#^ *p* < .10, * *p* < .05, ** *p* < .01

## Sample M*plus* code for the models assuming single-item DIF with adaptability as covariate (linear and quadratic effects; adopted from Bauer, 2016)

TITLE: MNLFA with factor mean and variance dependent

on covariates (including quadratic effects) – Single-item DIF for DE1

DATA: FILE IS StudentQuest.dat;

VARIABLE:

NAMES ARE

STUDID ! Student ID

SCHOOLID ! School ID

IMMIG ! Immigration status

CB ! Cognitive-behavioral adaptability

AE ! Affective-emotional adaptability

DE1-DE4; ! Epistemological beliefs items

USEVARIABLES ARE

DE1-DE4

CBc

CBcsq;

CATEGORICAL ARE

DE1-DE4;

MISSING ARE ALL(-99); ! Missing values are coded as -99

CLUSTER = SCHOOLID; ! Student data are nested in schools

CONSTRAINT = CBc

CBcsq; ! Further constraints specified for later

DEFINE: CBc = CB-2.9860; ! Mean-centered predictor

CBcsq = CBc**2; ! Create the quadratic term

ANALYSIS:

TYPE = COMPLEX; ! Adjust standard errors and chi-square statistics

ESTIMATOR = MLR; ! Specifies the Graded Response Model (GRM)

LINK = LOGIT; ! Logistic link function to make it a GRM

H1ITERATIONS = 10000;

MODEL:

! Starting values were obtained from the model without single-item DIF

! This model simply contained the linear and quadratic covariate effects

! on the latent variable (DE)

! Measurement model for DE

DE BY DE1*1.62337;

DE BY DE2*2.89672;

DE BY DE3*1.88120;

DE BY DE4*3.14895;

! Item thresholds

[DE1$1*-5.48020];

[DE1$2*-3.95711];

[DE1$3*-1.36587];

[DE1$4*0.29700];

[DE1$5*1.93687];

[DE2$1*-10.06147];

[DE2$2*-7.37973];

[DE2$3*-3.65769];

[DE2$4*-1.48709];

[DE2$5*1.33925];

[DE3$1*-6.91611];

[DE3$2*-4.88146];

[DE3$3*-2.54909];

[DE3$4*-1.13163];

[DE3$5*0.50628];

[DE4$1*-10.88013];

[DE4$2*-7.83573];

[DE4$3*-3.81768];

[DE4$4*-1.42170];

[DE4$5*1.31938];

! Factor mean

[DE@0];

! Name factor variance

DE(v_de);

! Regression of factor mean

DE ON CBc*0.76930;

DE ON CBcsq*0.02595;

! DIF item DE1

DE BY DE1(L); ! Factor loading is named ‘L’

DE1 ON CBc CBcsq; ! Regression of ‘intercept’ on covariates

MODEL CONSTRAINT :

! Define regression coefficients for factor variance

NEW (v_de1*-0.17106);

NEW(v_de2*0.18561);

! Regression of factor variance with exponential function as the link

v_de = EXP(v_de1*CBc + v_de2*CBcsq);

! Define regression coefficients for item factor loading

NEW(L0*0); NEW(L1*0); NEW(L2*0);

! Regression of item factor loading on covariates

L = L0 + L1*CBc + L2*CBcsq;

## Parameters of MNLFA Models with Immigration Status as Covariate

| *Covariate effects on parameters* | *Models assuming single-item DIF across immigration status (1 = Immigration status, 0 = Native Norwegian)* | | | |
| --- | --- | --- | --- | --- |
| DE factor |  |  |  |  |
| Mean | -0.20 (0.08)* | -0.15 (0.09) | -0.17 (0.09)* | -0.15 (0.09)^#^ |
| Variance^a^ | -0.18 (0.14) | -0.11 (0.15) | -0.11 (0.16) | -0.23 (0.17) |
|  |  |  |  |  |
| Item DE1 |  |  |  |  |
| Factor loading | 0.06 (0.15) | – | – | – |
| Intercept | 0.38 (0.17)* | – | – | – |
|  |  |  |  |  |
| Item DE2 |  |  |  |  |
| Factor loading | – | -0.27 (0.25) | – | – |
| Intercept | – | -0.31 (0.21) | – | – |
|  |  |  |  |  |
| Item DE3 |  |  |  |  |
| Factor loading | – | – | -0.33 (0.23) | – |
| Intercept | – | – | 0.02 (0.20) | – |
|  |  |  |  |  |
| Item DE4 |  |  |  |  |
| Factor loading | – | – | – | 0.51 (0.36) |
| Intercept | – | – | – | -0.09 (0.26) |
|  |  |  |  |  |
| *Model fit information* |  |  |  |  |
| LL | -7599.3 | -7601.1 | -7600.6 | -7600.0 |
| Npar | 28 | 28 | 28 | 28 |
| SCF | 1.1832 | 1.1920 | 1.1995 | 1.2055 |
| AIC | 15254.6 | 15258.1 | 15257.2 | 15256.0 |
| BIC | 15404.5 | 15408.0 | 15407.2 | 15405.9 |
| aBIC | 15315.6 | 15319.1 | 15318.2 | 15316.9 |

*Note.* DE = Epistemological beliefs in the development of scientific knowledge, LL = Loglikelihood value, Npar = Number of free parameters, SCF = Scaling correction factor, aBIC = Sample-size adjusted BIC. Statistically significant covariate effects are shaded in grey. ^a^A log-linear model was specified for the factor variance, variance = exp(*covariate*). Standard errors are shown in brackets. ^#^ *p* < .10, * *p* < .05, ** *p* < .01

## Parameters of MNLFA Models with Cognitive-Behavioral Adaptability and Immigration Status as Covariates

*E1) Interaction effects models for item DE1 (unstandardized)*

| *Covariate effects on parameters* | *CB* | *IMMIG* | *CB^2^* | *IMMIG×CB* | *IMMIG×CB^2^* |
| --- | --- | --- | --- | --- | --- |
| DE factor |  |  |  |  |  |
| Mean | 0.71 (0.05)* | -0.21 (0.11)^#^ | 0.01 (0.03) | 0.06 (0.11) | 0.01 (0.08) |
| Variance^a^ | -0.23 (0.07)** | -0.35 (0.15)* | 0.14 (0.05)** | 0.33 (0.15)* | 0.13 (0.07)^#^ |
|  |  |  |  |  |  |
| Item DE1 |  |  |  |  |  |
| Factor loading | -0.06 (0.09) | 0.02 (0.20) | 0.00 (0.04) | -0.16 (0.17) | -0.05 (0.07) |
| Intercept | 0.29 (0.07)** | 0.50 (0.19)* | 0.12 (0.06)* | -0.12 (0.22) | -0.09 (0.19) |
|  |  |  |  |  |  |
| *Model fit information* |  |  |  |  |  |
| LL | -7292.7 | | | | |
| Npar | 44 | | | | |
| SCF | 1.0928 | | | | |
| AIC | 14673.4 | | | | |
| BIC | 14908.7 | | | | |
| aBIC | 14768.9 | | | | |

*Note.* CB = Cognitive-behavioral adaptability score, IMMIG = Immigration status (1 = Immigration status, 0 = Native Norwegian), DE = Epistemological beliefs in the development of scientific knowledge, LL = Loglikelihood value, Npar = Number of free parameters, SCF = Scaling correction factor, aBIC = Sample-size adjusted BIC. Statistically significant covariate effects are shaded in grey. ^a^A log-linear model was specified for the factor variance, variance = exp(*covariate*). Standard errors are shown in brackets. ^#^ *p* < .10, * *p* < .05, ** *p* < .01

*E2) Interaction effects models for item DE2 (unstandardized)*

| *Covariate effects on parameters* | *CB* | *IMMIG* | *CB^2^* | *IMMIG×CB* | *IMMIG×CB^2^* |
| --- | --- | --- | --- | --- | --- |
| DE factor |  |  |  |  |  |
| Mean | 0.78 (0.05)** | -0.11 (0.12) | 0.02 (0.03) | 0.02 (0.11) | -0.05 (0.08) |
| Variance^a^ | -0.25 (0.06)** | -0.29 (0.18) | 0.15 (0.05)** | 0.28 (0.14)* | 0.12 (0.07)^#^ |
|  |  |  |  |  |  |
| Item DE2 |  |  |  |  |  |
| Factor loading | 0.09 (0.13) | -0.21 (0.33) | -0.01 (0.06) | -0.04 (0.28) | -0.04 (0.14) |
| Intercept | -0.35 (0.12)** | -0.43 (0.28) | -0.07 (0.11) | 0.36 (0.32) | 0.20 (0.21) |
|  |  |  |  |  |  |
| *Model fit information* |  |  |  |  |  |
| LL | -7298.9 | | | | |
| Npar | 44 | | | | |
| SCF | 1.0979 | | | | |
| AIC | 14685.8 | | | | |
| BIC | 14921.1 | | | | |
| aBIC | 14781.3 | | | | |

*Note.* CB = Cognitive-behavioral adaptability score, IMMIG = Immigration status (1 = Immigration status, 0 = Native Norwegian), DE = Epistemological beliefs in the development of scientific knowledge, LL = Loglikelihood value, Npar = Number of free parameters, SCF = Scaling correction factor, aBIC = Sample-size adjusted BIC. Statistically significant covariate effects are shaded in grey. ^a^A log-linear model was specified for the factor variance, variance = exp(*covariate*). Standard errors are shown in brackets. ^#^ *p* < .10, * *p* < .05, ** *p* < .01

*E3) Interaction effects models for item DE3 (unstandardized)*

| *Covariate effects on parameters* | *CB* | *IMMIG* | *CB^2^* | *IMMIG×CB* | *IMMIG×CB^2^* |
| --- | --- | --- | --- | --- | --- |
| DE factor |  |  |  |  |  |
| Mean | 0.77 (0.05)** | -0.17 (0.12) | 0.03 (0.04) | 0.02 (0.11) | -0.02 (0.08) |
| Variance^a^ | -0.23 (0.07)** | -0.27 (0.17) | 0.15 (0.05) | 0.28 (0.15)^#^ | 0.13 (0.07)^#^ |
|  |  |  |  |  |  |
| Item DE3 |  |  |  |  |  |
| Factor loading | 0.02 (0.13) | -0.26 (0.28) | -0.01 (0.05) | -0.04 (0.19) | -0.17 (0.12) |
| Intercept | -0.20 (0.10)* | 0.10 (0.25) | -0.09 (0.08) | 0.60 (0.28)* | -0.09 (0.19) |
|  |  |  |  |  |  |
| *Model fit information* |  |  |  |  |  |
| LL | -7298.3 | | | | |
| Npar | 44 | | | | |
| SCF | 1.1448 | | | | |
| AIC | 14684.6 | | | | |
| BIC | 14920.0 | | | | |
| aBIC | 14780.2 | | | | |

*Note.* CB = Cognitive-behavioral adaptability score, IMMIG = Immigration status (1 = Immigration status, 0 = Native Norwegian), DE = Epistemological beliefs in the development of scientific knowledge, LL = Loglikelihood value, Npar = Number of free parameters, SCF = Scaling correction factor, aBIC = Sample-size adjusted BIC. Statistically significant covariate effects are shaded in grey. ^a^A log-linear model was specified for the factor variance, variance = exp(*covariate*). Standard errors are shown in brackets. ^#^ *p* < .10, * *p* < .05, ** *p* < .01

*E4) Interaction effects models for item DE4 (unstandardized)*

| *Covariate effects on parameters* | *CB* | *IMMIG* | *CB^2^* | *IMMIG×CB* | *IMMIG×CB^2^* |
| --- | --- | --- | --- | --- | --- |
| DE factor |  |  |  |  |  |
| Mean | 0.74 (0.05)** | -0.13 (0.11) | 0.03 (0.04) | 0.04 (0.11) | -0.03 (0.08) |
| Variance^a^ | -0.21 (0.07)** | -0.47 (0.19)* | 0.16 (0.05)** | 0.21 (0.16) | 0.07 (0.07) |
|  |  |  |  |  |  |
| Item DE4 |  |  |  |  |  |
| Factor loading | -0.12 (0.13) | 0.59 (0.49) | -0.06 (0.07) | 0.65 (0.48) | 0.46 (0.33) |
| Intercept | 0.18 (0.11)^#^ | -0.21 (0.34) | -0.01 (0.08) | -0.86 (0.39) | -0.01 (0.26) |
|  |  |  |  |  |  |
| *Model fit information* |  |  |  |  |  |
| LL | -7297.4 | | | | |
| Npar | 44 | | | | |
| SCF | 1.1255 | | | | |
| AIC | 14682.8 | | | | |
| BIC | 14918.2 | | | | |
| aBIC | 14778.4 | | | | |

*Note.* CB = Cognitive-behavioral adaptability score, IMMIG = Immigration status (1 = Immigration status, 0 = Native Norwegian), DE = Epistemological beliefs in the development of scientific knowledge, LL = Loglikelihood value, Npar = Number of free parameters, SCF = Scaling correction factor, aBIC = Sample-size adjusted BIC. Statistically significant covariate effects are shaded in grey. ^a^A log-linear model was specified for the factor variance, variance = exp(*covariate*). Standard errors are shown in brackets. ^#^ *p* < .10, * *p* < .05, ** *p* < .01

## Parameters of MNLFA Models with Affective-Emotional Adaptability and Immigration Status as Covariates

*F1) Interaction effects models for item DE1 (unstandardized)*

| *Covariate effects on parameters* | *AE* | *IMMIG* | *AE^2^* | *IMMIG×AE* | *IMMIG×AE^2^* |
| --- | --- | --- | --- | --- | --- |
| DE factor |  |  |  |  |  |
| Mean | 0.38 (0.04)** | -0.11 (0.10) | 0.05 (0.03)^#^ | 0.00 (0.09) | -0.07 (0.07) |
| Variance^a^ | -0.20 (0.05)** | -0.19 (0.15) | 0.16 (0.04)** | 0.04 (0.14) | -0.04 (0.08) |
|  |  |  |  |  |  |
| Item DE1 |  |  |  |  |  |
| Factor loading | -0.03 (0.06) | 0.08 (0.17) | 0.02 (0.04) | -0.24 (0.13)^#^ | -0.10 (0.06)^#^ |
| Intercept | 0.19 (0.07)** | 0.35 (0.21)^#^ | -0.02 (0.05) | 0.00 (0.17) | 0.05 (0.13) |
|  |  |  |  |  |  |
| *Model fit information* |  |  |  |  |  |
| LL | -7418.7 | | | | |
| Npar | 44 | | | | |
| SCF | 1.0972 | | | | |
| AIC | 14925.3 | | | | |
| BIC | 15160.6 | | | | |
| aBIC | 15020.8 | | | | |

*Note.* AE = Affective-emotional adaptability score, IMMIG = Immigration status (1 = Immigration status, 0 = Native Norwegian), DE = Epistemological beliefs in the development of scientific knowledge, LL = Loglikelihood value, Npar = Number of free parameters, SCF = Scaling correction factor, aBIC = Sample-size adjusted BIC. Statistically significant covariate effects are shaded in grey. ^a^A log-linear model was specified for the factor variance, variance = exp(*covariate*). Standard errors are shown in brackets. ^#^ *p* < .10, * *p* < .05, ** *p* < .01

*F2) Interaction effects models for item DE2 (unstandardized)*

| *Covariate effects on parameters* | *AE* | *IMMIG* | *AE^2^* | *IMMIG×AE* | *IMMIG×AE^2^* |
| --- | --- | --- | --- | --- | --- |
| DE factor |  |  |  |  |  |
| Mean | 0.43 (0.04)** | -0.03 (0.11) | 0.06 (0.03)^#^ | -0.01 (0.09) | -0.09 (0.07) |
| Variance^a^ | -0.20 (0.05)** | -0.09 (0.15) | 0.16 (0.04)** | -0.02 (0.13) | -0.08 (0.08) |
|  |  |  |  |  |  |
| Item DE2 |  |  |  |  |  |
| Factor loading | 0.00 (0.09) | -0.34 (0.32) | -0.01 (0.05) | 0.03 (0.26) | 0.09 (0.10) |
| Intercept | -0.28 (0.09)** | -0.48 (0.26) | -0.09 (0.05)^#^ | 0.03 (0.25) | 0.18 (0.14) |
|  |  |  |  |  |  |
| *Model fit information* |  |  |  |  |  |
| LL | -7423.0 | | | | |
| Npar | 44 | | | | |
| SCF | 1.0898 | | | | |
| AIC | 14934.0 | | | | |
| BIC | 15169.3 | | | | |
| aBIC | 15029.6 | | | | |

*Note.* AE = Affective-emotional adaptability score, IMMIG = Immigration status (1 = Immigration status, 0 = Native Norwegian), DE = Epistemological beliefs in the development of scientific knowledge, LL = Loglikelihood value, Npar = Number of free parameters, SCF = Scaling correction factor, aBIC = Sample-size adjusted BIC. Statistically significant covariate effects are shaded in grey. ^a^A log-linear model was specified for the factor variance, variance = exp(*covariate*). Standard errors are shown in brackets. ^#^ *p* < .10, * *p* < .05, ** *p* < .01

*F3) Interaction effects models for item DE3 (unstandardized)*

| *Covariate effects on parameters* | *AE* | *IMMIG* | *AE^2^* | *IMMIG×AE* | *IMMIG×AE^2^* |
| --- | --- | --- | --- | --- | --- |
| DE factor |  |  |  |  |  |
| Mean | 0.41 (0.04)** | -0.10 (0.10) | 0.05 (0.03)^#^ | -0.03 (0.09) | -0.06 (0.07) |
| Variance^a^ | -0.18 (0.05)** | -0.13 (0.16) | 0.15 (0.04)** | -0.04 (0.14) | -0.03 (0.08) |
|  |  |  |  |  |  |
| Item DE3 |  |  |  |  |  |
| Factor loading | -0.08 (0.08) | -0.06 (0.26) | 0.07 (0.04) | 0.16 (0.24) | -0.19 (0.13) |
| Intercept | -0.05 (0.05) | 0.21 (0.18) | 0.05 (0.06) | 0.51 (0.23)* | -0.16 (0.12) |
|  |  |  |  |  |  |
| *Model fit information* |  |  |  |  |  |
| LL | -7421.4 | | | | |
| Npar | 44 | | | | |
| SCF | 1.1584 | | | | |
| AIC | 14930.8 | | | | |
| BIC | 15166.1 | | | | |
| aBIC | 15026.4 | | | | |

*Note.* AE = Affective-emotional adaptability score, IMMIG = Immigration status (1 = Immigration status, 0 = Native Norwegian), DE = Epistemological beliefs in the development of scientific knowledge, LL = Loglikelihood value, Npar = Number of free parameters, SCF = Scaling correction factor, aBIC = Sample-size adjusted BIC. Statistically significant covariate effects are shaded in grey. ^a^A log-linear model was specified for the factor variance, variance = exp(*covariate*). Standard errors are shown in brackets. ^#^ *p* < .10, * *p* < .05, ** *p* < .01

*F4) Interaction effects models for item DE4 (unstandardized)*

| *Covariate effects on parameters* | *AE* | *IMMIG* | *AE^2^* | *IMMIG×AE* | *IMMIG×AE^2^* |
| --- | --- | --- | --- | --- | --- |
| DE factor |  |  |  |  |  |
| Mean | 0.41 (0.05)** | -0.06 (0.10) | 0.04 (0.03) | 0.01 (0.09) | -0.07 (0.06) |
| Variance^a^ | -0.21 (0.05)** | -0.23 (0.19) | 0.18 (0.04)** | -0.05 (0.14) | -0.11 (0.08) |
|  |  |  |  |  |  |
| Item DE4 |  |  |  |  |  |
| Factor loading | 0.09 (0.08) | 0.40 (0.43) | -0.09 (0.06) | 0.24 (0.28) | 0.29 (0.16)^#^ |
| Intercept | 0.11 (0.09) | -0.13 (0.34) | 0.05 (0.05) | -0.60 (0.25)* | -0.04 (0.14) |
|  |  |  |  |  |  |
| *Model fit information* |  |  |  |  |  |
| LL | -7420.6 | | | | |
| Npar | 44 | | | | |
| SCF | 1.1112 | | | | |
| AIC | 14929.2 | | | | |
| BIC | 15164.5 | | | | |
| aBIC | 15024.7 | | | | |

*Note.* AE = Affective-emotional adaptability score, IMMIG = Immigration status (1 = Immigration status, 0 = Native Norwegian), DE = Epistemological beliefs in the development of scientific knowledge, LL = Loglikelihood value, Npar = Number of free parameters, SCF = Scaling correction factor, aBIC = Sample-size adjusted BIC. Statistically significant covariate effects are shaded in grey. ^a^A log-linear model was specified for the factor variance, variance = exp(*covariate*). Standard errors are shown in brackets. ^#^ *p* < .10, * *p* < .05, ** *p* < .01

## References

Bauer, D. J. (2016). A More General Model for Testing Measurement Invariance and Differential Item Functioning. *Psychological Methods*. doi:10.1037/met0000077
